# Pulsed, continuous or somewhere in between? Resource dynamics matter in the optimisation of microbial communities

**DOI:** 10.1038/s41396-023-01369-1

**Published:** 2023-01-24

**Authors:** Andrew D. Letten, William B. Ludington

**Affiliations:** 1 School of Biological Sciences, University of Queensland, Brisbane, QLD 4072 Australia; 2 Department of Embryology, Carnegie Institution of Washington, Baltimore, MD USA; 3 Department of Biology, Johns Hopkins University, Baltimore, MD USA

**Keywords:** Microbial ecology, Microbiome, Theoretical ecology

## Abstract

The optimisation of synthetic and natural microbial communities has vast potential for emerging applications in medicine, agriculture and industry. Realising this goal is contingent on a close correlation between theory, experiments, and the real world. Although the temporal pattern of resource supply can play a major role in microbial community assembly, resource dynamics are commonly treated inconsistently in theoretical and experimental research. Here we explore how the composition of communities varies under continuous resource supply, typical of theoretical approaches, versus pulsed resource supply, typical of experiments. Using simulations of classical resource competition models, we show that community composition diverges rapidly between the two regimes, with almost zero overlap in composition once the pulsing interval stretches beyond just four hours. The implication for the rapidly growing field of microbial community optimisation is that the resource supply regime must be tailored to the community being optimised. As such, we argue that resource supply dynamics should be considered both a constraint in the design of novel microbial communities and as a tuning mechanism for the optimisation of pre-existing communities like those found in the human gut.

There is a growing impetus to leverage our fundamental understanding of microbial community assembly towards applied problems. With microbes contributing to diverse physiological, biogeochemical, and agricultural processes, the potential to control and optimise microbial communities holds promise for interventions ranging from industrial and environmental remediation to human medicine and biofuel production [1, 2]. Realising this goal is contingent on high fidelity between theory, experiments, and the natural dynamics of target systems.

Theoretical and experimental research in microbial community optimisation has largely proceeded along two parallel paths. Theoretical approaches leverage mathematical models and metabolic networks to predict which species combinations are stable and how they can optimise a given function (e.g., maximum biomass, waste degradation or host health) [3–7]. Experimental studies often take a combinatorial approach, iteratively assembling different species combinations in vitro and evaluating their stability and functional attributes [8–11]. Both theory and experiments are valuable but they are also susceptible to their own modus operandi that may limit their correspondence and their translation to real-world systems. On the one hand, theoretical approaches typically adopt the analytical tractability of steady state dynamics, where microbial consumers and the resources on which they depend are assumed to establish a stable equilibrium. On the other hand, experimental approaches almost exclusively embrace the high-throughput efficiency of serial-batch culture, where consumers and resources are made to fluctuate over several orders of magnitude with each serial passage. This raises an important question: should we expect unity in the composition of optimised communities emerging under continuous resource supply (e.g., chemostat) versus the discrete pulsed resource supply of, for example, serial-batch culture?

To explore how microbial community composition varies under contrasting resource supply dynamics, we performed simulations of a classical resource-competition model:

1 $$\frac{{dN_i}}{{dt}} = N_i\left( {\mathop {\sum}\limits_{j = 1}^n {\mu _{ij}\left( {R_j} \right) - m} } \right)$$

2 $$\frac{{dR_j}}{{dt}} = {\Psi}_j\left( {R_j} \right) - \mathop {\sum }\limits_{i = 1}^n Q_{ij}\mu _{ij}\left( {R_j} \right)N_i,$$

where *N*_*i*_ is the population density of consumer *i*, *R*_*j*_ is the concentration of resource *j*, *μ*_*ij*_(*R*_*j*_) is the per capita functional response of consumer *i*, *m* is the per capita mortality rate due to dilution, *Ψ*_*j*_(*R*_*j*_) is the resource supply function, and *Q*_*ij*_ is the resource quota of consumer *i* on resource *j* (amount of resource per unit consumer). The consumer functional response is given by the Monod function, $\mu _{ij}(R_j) = \mu _{max_{ij}}\frac{{R_j}}{{K_{s_{ij}} + R_j}}$ , where $\mu _{max_{ij}}$ is the maximum growth rate and $K_{s_{ij}}$ is the half saturation constant for consumer *i* on resource *j*.

To set up the simulations, we randomly sampled the parameters of the Monod growth functions, (*μ*_*max*_ and *K*_*s*_) for five species competing for five substitutable resources (essential resources are treated separately in the supplementary information, with similar findings). In one set of parametrisations (*n* = 100 unique competitor combinations) we used both random *μ*_*max*_ and *K*_*s*_, and in another set (*n* = 100) we imposed a trade-off in maximum growth rate and substrate affinity $( {\frac{{\mu _{max}}}{{K_s}}} )$ (Fig. 1a). The rationale for imposing a trade-off is that metabolic theory predicts that organisms that invest energy into a high maximum growth rate will have lower substrate affinities and vice versa [12, 13]. To ensure reasonable growth rates relative to the time-scale of resource pulsing, we sampled *μ*_*max*_ such that minimum doubling times spanned from 21 to 52 min (when all resources are non-limiting). For each of the random competitor combinations, we simulated resources under continuous or pulsed resource supply with resource replenishment every 1/2, 1, 2, 4, 12, or 24 h. Under pulsed resource supply, *Ψ*_*j*_(*R*_*j*_) and *m* are removed from Eq. (1) and (2) and replaced by discontinuous resource pulsing and cell transfer at fixed intervals. The total resource flux (and mortality) was held constant under all frequencies of resource supply i.e., less frequent replenishment corresponds to larger resource pulses (see Supplementary Information for full model/simulation specifications).

**Fig. 1:**
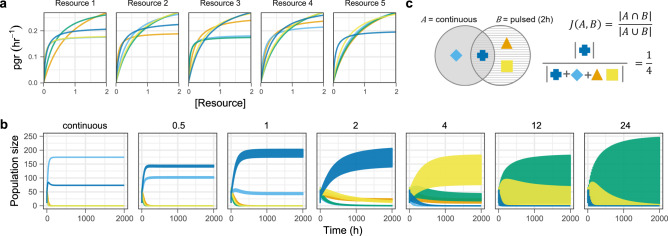
Quantifying compositional overlap between communities assembled under continuous vs. pulsed resource supply. **a** Per capita growth responses (Monod functions) from a single iteration of the model assuming a trade-off between maximum growth rate and resource affinity (colours correspond to individual consumers). **b** Time series of consumers in **a** under different resource supply regimes. Numbers above individual panels reflect pulsing intervals in hours. The amplitude of population fluctuations increases with longer intervals between pulses, with distinct phases of growth, saturation, and instantaneous mortality visible at a finer temporal resolution (Fig. S10). **c** Example measure of compositional overlap (Jaccard similarity index) between communities assembled under continuous resource supply (far left panel in **b**) vs. pulsing every two hours (centre panel in **b**).

After allowing the competitors to reach a steady state (time-averaged over 24 h under pulsed treatments), we quantified the correspondence between the continuous supply treatment and the pulsed treatments using the Jaccard similarity index, $J\left( {A,B} \right) = \frac{{\left| {A \cap B} \right|}}{{\left| {A \cup B} \right|}}$ (0 ≤ *J*(*A*,*B*) ≤ 1), where the numerator gives the number of species (max = 5) that persist under continuous (*A*) *and* pulsed (*B*) resource supply, and the denominator gives the number of species (max = 5) that persist under continuous *or* pulsed resource supply (Fig. 1b, c).

Under both sets of simulations (with and without enforcing a trade-off between maximal growth rate and resource affinity), we observe that the similarity in final community composition between continuous and pulsed resource supply decays with increasingly large intervals between resource replenishment (Fig. 2a). When no trade-off is imposed between maximum growth rate and resource affinity (orange line in Fig. 2a) the mean compositional similarity is only 0.68 when resources are pulsed every 2 h and down to 0.41 when resources are pulsed every 24 h (typical of serial-batch culture). The rate of decay in the Jaccard index is more severe when a trade-off is imposed between maximum growth rate and substrate affinity, to the extent that once pulsing intervals reach four hours there is almost zero overlap in community composition (blue line in Fig. 2a).

**Fig. 2:**
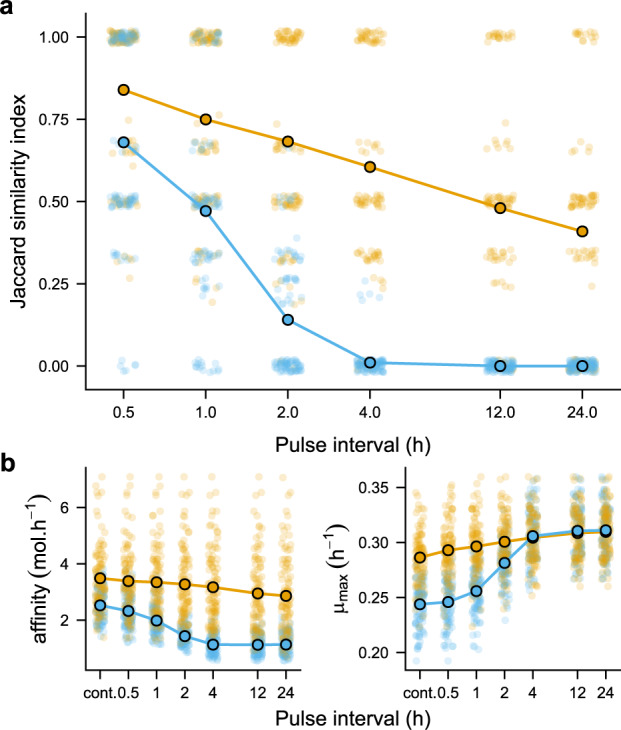
Impact of resource supply regime on community composition and abundance weighted mean trait values. **a** Compositional overlap (Jaccard similarity) between communities under continuous versus pulsed resource supply. Orange lines, points and circles denote model parametrisations with random sampling of both *μ*_*max*_ and *K*_*s*_; blue lines, points and circles denote model parametrisations with a trade-off imposed between *μ*_*max*_ and resource affinity $( {\frac{{\mu _{max}}}{{K_s}}} )$. Simulation parameters provided in the Supplementary Information. **b** Mean trait values for affinity and *μ*_*max*_ averaged for each consumer across the five resources and weighted by their final abundance at the end of a simulation (cont. = continuous). In both **a** and **b**, small points (jittered for clarity) give the result of an individual simulation; large circles indicate the corresponding mean.

Ecological theory provides an intuitive explanation for these observations. When resources are more continuously supplied, the better competitor is the one that can sustain a positive growth rate at the lowest concentrations of a limiting resource (i.e., has a higher resource affinity or lower R^*^ in the language of resource competition theory [14]). In contrast, under increasingly pulsed resource supply, the better competitor is the one that can grow rapidly at higher resource concentrations. Having a high resource affinity (low R^*^) is of little benefit if resource concentrations fluctuate over large amplitudes because it only confers an ephemeral competitive advantage in the brief period before the resource is completely depleted (ahead of the next resource pulse). Instead, a high maximum growth rate is optimal because it allows the consumer to grow rapidly and quickly deplete a shared limiting resource. This high maximum growth strategy is, however, sub-optimal under continuous resource supply because a low R^*^ strategist can draw the resource down and hold it at a concentration at which the maximum growth strategist is unable to maintain a positive growth rate.

Looking at the mean trait values for resource affinity and *μ*_*max*_ weighted by each consumer’s final abundance, it is indeed apparent that consumers with a higher affinity (averaged across the five resources) are favoured under continuous resource supply, while consumers with high maximum growth rates are favoured under pulsing intervals of increasing length (Fig. 2b). Enforcing this trade-off, therefore, leads to the rapid decline in compositional similarity we observe under resource pulsing. Notably, it also leads to a richness peak at intermediate pulsing intervals, where these alternative strategies have a higher probability of coexisting [15] (Fig. S1). At the same time, we still observe a decline in compositional similarity when *μ*_*max*_ and *K*_*s*_ are randomly sampled independently of each other simply because the trade-off between maximum growth and resource affinity will emerge occasionally by chance. Two experimental tests of microbial community composition under continuous versus pulsed resource supply are consistent with these observations [16, 17].

To evaluate the sensitivity of these observations to different assumptions, we ran additional simulations under various alternative model parameterisations and formulations. In brief, comparable trends to those described above are observed when: i) maximum growth rates are faster or slower than those presented in the main text (Figs. S2, S3); ii) all resources are assumed to be essential to growth (following Liebig’s law of the minimum) (Fig. S4); iii) a weaker trade-off is imposed between maximum growth and affinity (Figs. S5, S6); or iv) mortality is continuous rather than intermittent (Figs. S7, S8). We also investigated the relationship between observed compositional overlap and the dynamical stability under continuous resource supply, anticipating that more stable communities would tend to be more resistant to compositional shifts under resource pulsing. The reality appears more nuanced, namely that weaker dynamical stability at the limit of constant resource supply is associated with higher variance in compositional overlap under continuous vs. pulsed conditions (Fig. S9). In other words, systems with weaker stability are less predictable. A wide range of other microbial traits and trade-offs may interact unpredictably with the relationship between resource supply and community composition. The potential modulating role of system instabilities generated by cross-feeding interactions, non-convex trade-off functions, and the evolution of specialist versus generalist strategies present several especially valuable lines of enquiry [18–20].

Although these observations are germane to any consumer-resource system, our emphasis here is on the emerging field of microbial community optimisation, where the practical implications are especially timely and important; namely, the resource supply regime must be tailored to the community being optimised. For example, wastewater treatment might be more appropriately modelled under continuous resource supply [21], whereas fermented food and beverage production may be more closely allied to the pulsed resource dynamics observed in batch culture [22]. Resource supply might also be manipulated to favourably modify the competitive hierarchy in an existing community (e.g., by regulating the rate of nutrient supply to the gut through meal timing). Indeed, there is emerging evidence that feeding frequency can drive significant changes in gut microbiota composition [23, 24]. Thus, resource supply dynamics should be considered both a constraint in the design of novel microbial communities and as a tuning mechanism for the optimisation of preexisting communities like those found in the human gut.

## Supplementary information

Supplementary Information

## Data Availability

No datasets were generated or analysed as part of the current study.

## Code availability

R and Python code to reproduce simulations and analysis available at: https://github.com/andrewletten/respulse-ISMEJ.
