# Supplementary material for: Pulsed, continuous or somewhere in between? Resource dynamics matter in the optimisation of microbial communities
Source: ISME J. 2023 Jan 24;17(4):641–4. doi: 10.1038/s41396-023-01369-1 (PMC10030971; doi:10.1038/s41396-023-01369-1)
Supplement: Supplementary file 1 — Supplementary Information [file 41396_2023_1369_MOESM1_ESM.pdf]

Supplementary Information:
Pulsed, continuous or somewhere in between? Resource dynamics matter in the optimization of microbial communities

Andrew D. Letten*[1] and William B. Ludington[2,3]

[1]School of Biological Sciences, University of Queensland, Brisbane, Queensland 4072, Australia
[2]Department of Embryology, Carnegie Institution of Washington, Baltimore, MD, United States
[3]Department of Biology, Johns Hopkins University, Baltimore, MD, United States

January 12, 2023

*Correspondence author. Email: a.letten@uq.edu.au

SI-1: Model formulation, parameterisation and simulation

All simulation models take the general form:

$$\frac{\mathrm{d}N_i}{\mathrm{d}t} = N_i(\mu_i(R_1, R_2, ...) - m)\,, \tag{S1}$$

$$\frac{\mathrm{d}R_j}{\mathrm{d}t} = \Psi_j(R_j) - \sum_{i=1}^{n} Q_{ij}\mu_{ij}(R_j)N_i\,, \tag{S2}$$

where N_i is the population density of consumer i, R_j is the concentration of resource j, $\mu_i(R_j)$ is the per capita consumer functional response of consumer i, m is the density-independent mortality rate due to dilution, $\Psi_j(R_j)$ is the resource supply function, and Q_{ij} is the resource quota of consumer i on resource j (amount of resource per unit consumer). Resources are assumed to be substitutable, with

$$\mu_i(R_1, R_2, ...) = \sum_{j=1}^{n} \mu_{ij}(R_j). \tag{S3}$$

The consumer functional response is given by the Monod function,

$$\mu_{ij}(R_j) = \mu_{max_{ij}} \frac{R_j}{K_{s_{ij}} + R_j}\,, \tag{S4}$$

where $\mu_{max_{ij}}$ is the maximum growth rate and $K_{s_{ij}}$ is the half saturation constant for consumer i on resource j.

Under chemostat dynamics, the resource supply function is given by $\Psi_j(R_j) = m(S_j - R_j)$, where m is the dilution rate and S_j is the supply concentration of resource j. Under pulsed resource supply, $\Psi_j(R_j)$ and m are removed from Eqs S1 and S2 and replaced by discontinuous resource/cell pulsing/transfer at fixed intervals:

$$R_j(t^+) = (1-d)(S_j) - dR_j(t^-), t = k\tau, k = 1, 2, ..., \tag{S5}$$

$$N_i(t^+) = dN_i(t^-), t = k\tau, k = 1, 2, ..., \tag{S6}$$

where τ is the pulse period (i.e., 0.5, 1, 2, 4, 12, or 24 hours) and d is the transfer fraction (the fraction of cells and resources carried over). $R_j(t^+)$ & $N_i(t^+)$ and $R_j(t^-)$ & $N_i(t^-)$ represent the respective right and left limits immediately following and preceding a pulse/transfer at time t. To approximate equivalent total resource flux (and mortality) under resource pulsing, (i.e., longer intervals correspond to a larger fraction of resource replenishment), d is given by $e^{-m\tau}$[1].

For all simulations, the Monod growth parameters (μ_{max} and K_s) were obtained by first sampling a set of values, S, from a uniform distribution ($U[0.4, 1.0]$) and transforming the set based on the following criteria. For all simulations described in the main text, $\mu_{max} = 0.4S$ (cf. *Alternative model formulations/parameterisations* described below). This equates to maximum growth rates of $0.16 - 0.4\text{h}^{-1}$, or minimum doubling times of 21-52 minutes when resources are non-limiting. In the set of simulations imposing a trade-off between maximum growth rate and affinity, $K_s = S^4$. Because affinity $= \frac{\mu_{max}}{K_s}$, $K_s = S^a$ gives a trade-off for any exponent $a > 1$. A choice of $a = 4$ is somewhat arbitrary in the absence of robust empirical data, however, we note that a weaker trade-off, given by $a = 2$, results counter-intuitively in a more rapid decay in compositional overlap (SI-3, Fig S5a). In the set of simulations where K_s is sampled independently of μ_{max}, a new set of values, S' was drawn from U[0.4, 1.0], with $K_s = S'^4$ to ensure a direct comparison with simulations imposing a tradeoff.

In all simulations $Q_{ij} = 0.1$ (units of resource), $S_j = 5$ (units of resource), and $m = 0.25\text{h}^{-1}$. Simulations were run with a fixed time step of 0.1h (dt = 0.1) for 4000 "hours" and initial densities/concentrations of 10 and 5 for all consumers and resources respectively. Any consumers whose populations reached above 0.01 in the final 100 time steps were treated as persisting. All models were simulated with the rescomp package (v0.1.0)[2] in R (version 4.1.2).

SI-2: Consumer richness

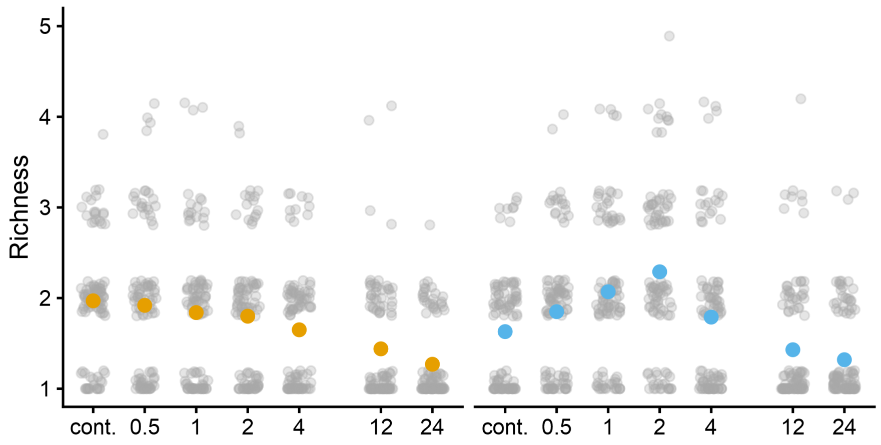

Figure S1: Consumer richness at steady state (time-averaged under pulsing) for all simulations presented in the main text. Grey points (jittered for clarity) give the result of an individual simulation; coloured circles indicate the corresponding mean. Orange circles denote model parametrisations with random sampling of both μ_{max} and K_s; blue lines, points and circles denote model parametrisations with a trade-off imposed between μ_{max} and resource affinity ($\frac{\mu_{max}}{K_s}$).

SI-3: Alternative model formulations/parameterisations

Faster/slower maximum growth rates

In the main results, consumer maximum growth rates were constrained between 0.16h^{-1} and 0.4h^{-1}. Here, we present comparative results when maximum growth rates are slower ($\mu_{max} = 0.2S$ [minimum doubling time: 42-104 min], $\mu_{max} = 0.1S$ [minimum doubling time: 83-208 min]) or faster ($\mu_{max} = S$ [minimum doubling time: 8-21 min]) than those presented in the main text. The motivation for considering alternative growth rates is that the slower the population growth rate is relative to the resource pulsing schedule, the closer the resource dynamic becomes to approximating a continuous supply (relative to the generation time).

Fig S2 corresponds to Figure 2 from the main text for the three alternative μ_{max} ranges (Fig S2a,c&d) alongside the original figure for reference (Fig S2b). Most notably the trend in decreasing community overlap is consistent across all four ‘treatments’, and it is only when the maximum doubling time extends beyond three hours that we observe a marked reduction in the dissimilarity between continuous and pulsed resource conditions (Fig S2d). The corresponding abundance weighted trait means are shown in Figure S3.

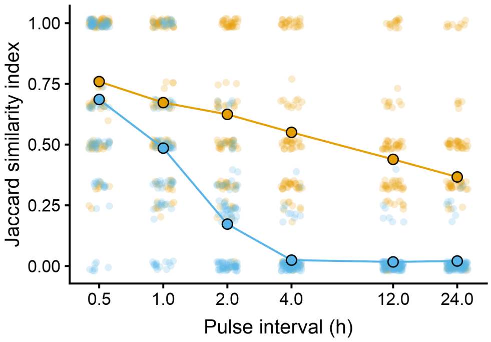

(a) Minimum doubling time: 8-21 mins

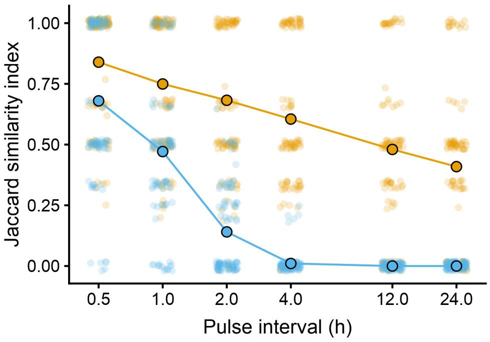

(b) Minimum doubling time: 21-52 mins

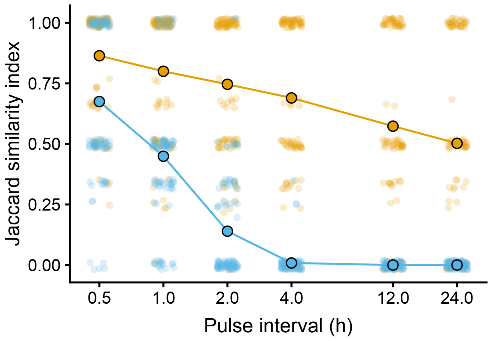

(c) Minimum doubling time: 42-104 mins

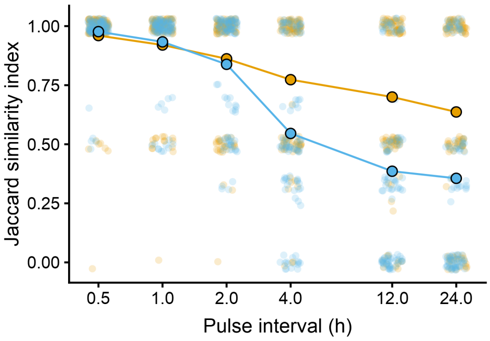

(d) Minimum doubling time: 83-208 mins

Figure S2: Compositional overlap (Jaccard similarity) between communities under continuous versus pulsed resource supply. Minimum doubling times (when all resources are non-limiting) of a) 8-21 mins, b) 21-52 mins (as shown in the main text) c) 42-104 mins, and d) 83-208 mins. Orange lines, points and circles denote model parametrisations with random sampling of both μ_{max} and K_s; blue lines, points and circles denote model parametrisations with a trade-off imposed between μ_{max} and K_s. Small points (jittered for clarity) give the result of an individual simulation; large circles indicate the corresponding mean.

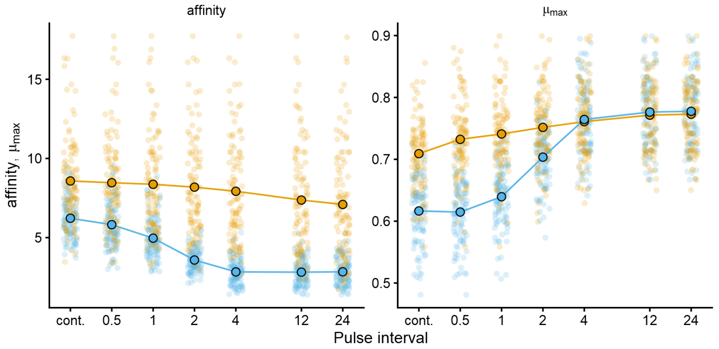

(a) Minimum doubling time: 8-21 mins

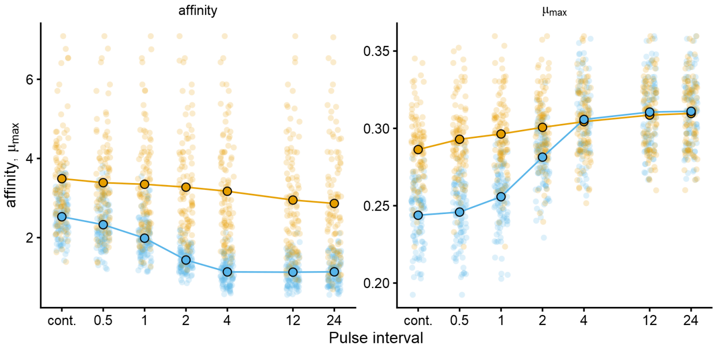

(b) Minimum doubling time: 21-52 mins

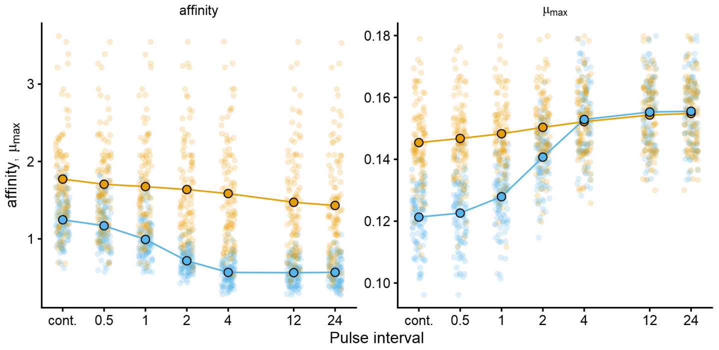

(c) Minimum doubling time: 42-104 mins

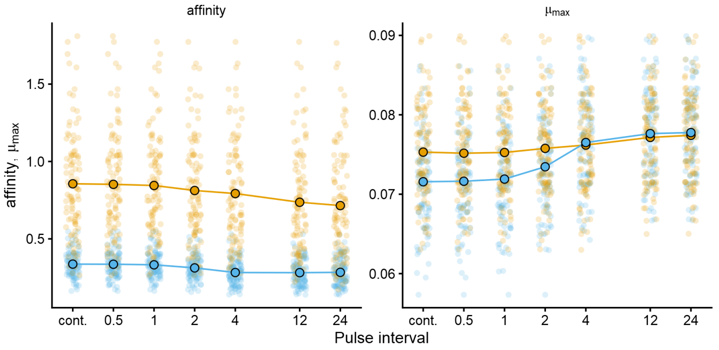

(d) Minimum doubling time: 83-208 mins

Figure S3: Mean trait values for affinity and μ_{max} averaged for each consumer across the five resources and weighted by their final abundance at the end of a simulation. Minimum doubling times (when all resources are non-limiting) of a) 8-21 mins, b) 21-52 mins (as shown in the main text) c) 42-104 mins, and d) 83-208 mins. Orange lines, points and circles denote model parametrisations with random sampling of both μ_{max} and K_s; blue lines, points and circles denote model parametrisations with a trade-off imposed between μ_{max} and K_s. Small points (jittered for clarity) give the result of an individual simulation; large circles indicate the corresponding mean.

Essential resources

The simulations described in the main text assume that the resources being consumed are substitutable, for example different carbon sources. Here we consider resources that are each essential to growth following Liebig's law of the minimum (e.g., carbon and nitrogen), in which case the first term in the parentheses in Equation S1 must be modified such that:

$$\mu_i(R_1, R_2, ...) = min(\mu_{i1}(R_1), \mu_{i2}(R_2), ...). \tag{S7}$$

All simulations of competition for essential resource were conducted as in the main text, except for: i) making $\mu_{max} = S$ (rather than $\mu_{max} = 0.4S$) to account for the realised growth rate being set by the most limiting resource; and ii) making the resource quotas proportional to the inverse of resource affinity, $Q_{ij} = 0.1(\frac{K_s}{\mu_{max}})$. Following optimal foraging theory, organisms should prioritise consumption of resource that are most beneficial to their growth [3, 4]. For substitutable resources this is the resource for which the consumer is a better competitor at equilibrium (i.e., has a lower R^*). For the core simulations, this was achieved by setting all Q to 0.1, which has the effect that consumption rates are determined solely by growth rate at equilibrium. For essential resources, it is the resource that the consumer is most limited by at equilibrium (i.e., has a lower affinity), hence the rationale for making the resource quota proportional to the inverse of resource affinity.

As for substitutable resources, under competition for essential resources we observe a similar, albeit slightly less sharp, monotonic decay in compositional overlap between the continuous resource supply and the pulsed resource treatments with increasingly large intervals between resource replenishment (Fig S4).

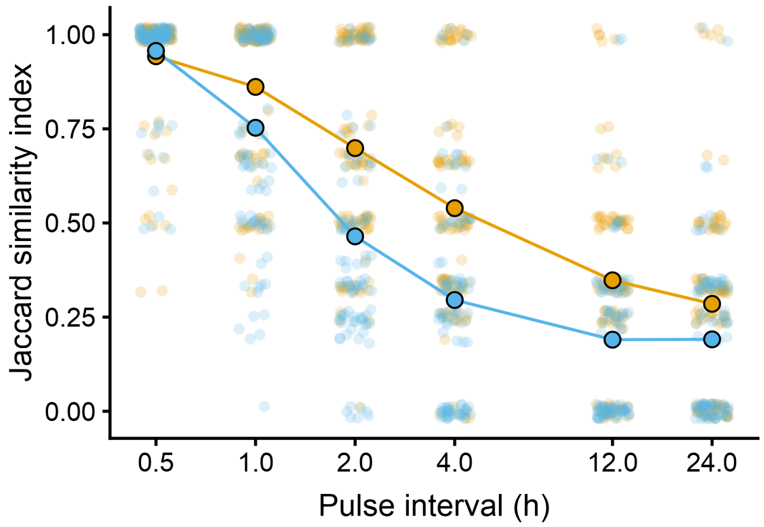

Figure S4: Compositional overlap (Jaccard similarity) between communities under continuous versus pulsed supply of **essential resources**. Orange lines, points and circles denote model parametrisations with random sampling of both μ_{max} and K_s; blue lines, points and circles denote model parametrisations with a trade-off imposed between μ_{max} and resource affinity ($\frac{\mu_{max}}{K_s}$). Small points (jittered for clarity) give the result of an individual simulation; large circles indicate the corresponding mean.

Weaker trade-off between μ_{max} and affinity

As noted under SI-1, for simulations imposing a trade-off between maximum

growth rate and affinity, we set $K_s = S^a$ where $a = 4$. Here we provide comparative results when $a = 2$ (weaker trade-off) or $a = 1$ (no trade-off; all consumers have identical resource affinity). When $a = 2$, we observe a more rapid decay in compositional similarity than observed when $a = 4$ (Fig. S5a). Our intuition is that in the absence of a stronger trade-off, there is less coexistence of alternative strategies at intermediate pulsing intervals (cf. simulations parameterised with $a = 4$), which translates into more rapid turnover of consumers across the spectrum of pulsing intervals. When $a = 1$ we observe compositional overlap much closer to unity overall (Fig. S6a), which is to be expected given all consumers have identical resource affinity when $a = 1$ (Fig. S6b). Nevertheless, it is notable that compositional overlap at 24h (79%) is still less than that observed at 2h (86%). The implication is that even in the absence of trade-offs along individual resource axes (including those that would emerge by chance when K_s is sampled independently of μ_{max}), higher-dimensional trade-offs can still emerge given competition for multiple resources. Note that by making $K_s = S^a$, we assume a convex relationship between affinity and maximum growth rate, which favours specialization. In contrast, a concave trade-off function will tend to favour generalist, intermediate strategies [5]. As such, given a concave trade-off function, we might expect generalists to be selected for over a broad range of intermediate pulsing intervals, with specialists only appearing at the extremes.

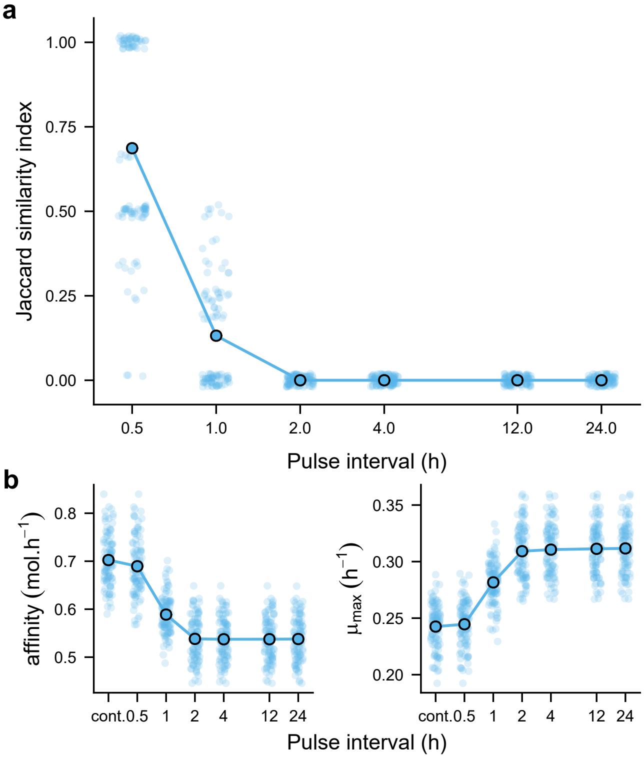

Figure S5: Impact of resource supply regime on community composition and adundance weighted mean trait values. K_s is equal to S^2 (i.e., proportional to the square of μ_{max}) (all other parameters as described in SI-1.) **(a)** Compositional overlap (Jaccard similarity) between communities under continuous versus pulsed resource supply. **(b)** Mean trait values for affinity and μ_{max} averaged for each consumer across the five resources and weighted by their final abundance at the end of a simulation. In both **(a)** and **(b)**, small points (jittered for clarity) give the result of an individual simulation; large circles indicate the corresponding mean.

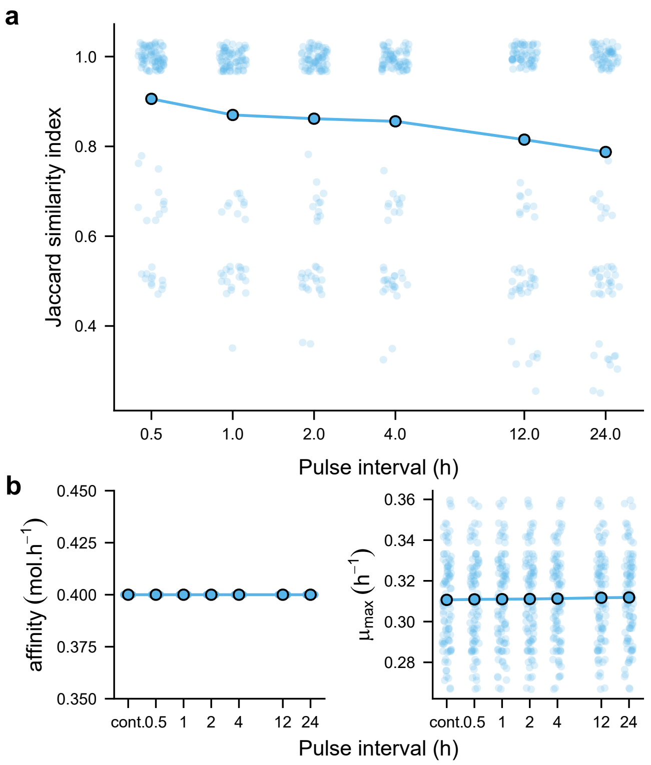

Figure S6: Impact of resource supply regime on community composition and adundance weighted mean trait values. K_s is equal to S (i.e., directly proportional to μ_{max}) (all other parameters as described in SI-1.) **(a)** Compositional overlap (Jaccard similarity) between communities under continuous versus pulsed resource supply. **(b)** Mean trait values for affinity and μ_{max} averaged for each consumer across the five resources and weighted by their final abundance at the end of a simulation. In both **(a)** and **(b)**, small points (jittered for clarity) give the result of an individual simulation; large circles indicate the corresponding mean.

Continuous mortality

Experimental serial transfer typically involves culturing cells in batch before transferring a small fraction of cells from the current batch to fresh media after a certain amount of time has elapsed. As such, it is characterised not just by pulses in resource supply but also significant instantaneous "mortality" corresponding to the substantial fraction of cells that are left behind. To maintain fidelity to typical serial transfer designs, the core simulations described in the main text included these instantaneous changes in population size that occur in step with resource pulsing. To delineate the impact of resource pulsing from mortality "pulsing", here we consider a scenario where resources are pulsed but mortality is continuous. Although somewhat contrived, this might be seen as approximating a system in which there is periodic resource supply coupled with continuous outflow of cells.

To this end, we ran simulations identical to those described in the main text with the only difference being the treatment of mortality as continuous rather than discrete. As shown in Figures S7 and S8 (corresponding to Fig 2a and 1b from the main text), the simulation results are near identical (>97% agreement) to those observed with discrete mortality. The small number of simulations that differed in Jaccard scores between mortality treatments correspond to those in which one of the consumers hovered either side of the extinction cut-off of 0.001.

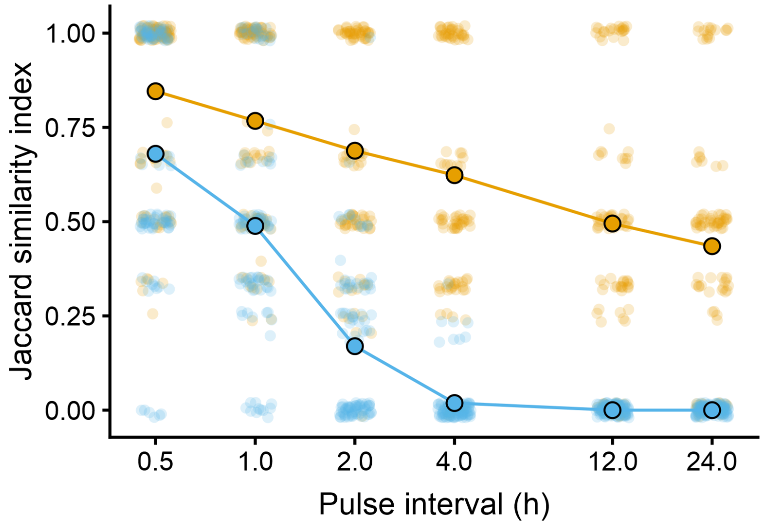

Figure S7: Compositional overlap (Jaccard similarity) between communities under continuous versus pulsed supply of resources (**with constant mortality**). Orange lines, points and circles denote model parametrisations with random sampling of both μ_{max} and K_s; blue lines, points and circles denote model parametrisations with a trade-off imposed between μ_{max} and resource affinity ($\frac{\mu_{max}}{K_s}$). Small points (jittered for clarity) give the result of an individual simulation; large circles indicate the corresponding mean. Simulation parameters provided in the Supplementary Information.

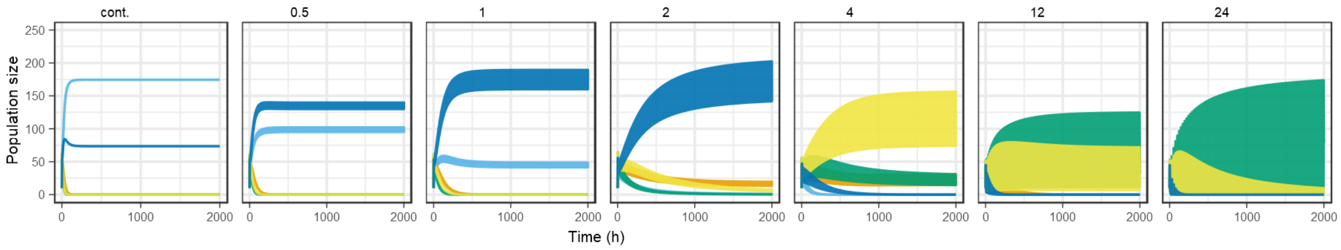

Figure S8: Time series of consumers in Fig 1a of main text under different resource supply regimes (**with constant mortality**). Numbers above individual panels reflect pulsing interval in hours (cont. = continuous).

SI-4: Compositional overlap as a function of dynamical stability

Here we consider the influence of dynamical stability at equilibrium in continuous culture on compositional overlap between communities assembled under continuous or pulsed resource supply. To this end, for all model paramterisations described in the main text we determined the Jacobian matrix and evaluated it at the equilibrium given by the numerical solution under continuous culture, using SymPy 1.9 [6] in Python 3.6.13. Taking the leading eigenvalue of the Jacobian as a measure of (in)stability (leading eigenvalues negative for all 200 simulations indicating stable equilibriums), we then inspected the relationship between Jaccard similarity and dynamical stability at the limit of continuous resource supply (Fig. S9). As noted in the main text, the overarching observation is that variance in compositional overlap increases with decreasing stability, which in turn suggests that compositional overlap between pulsed and continuous conditions is less predictable in systems with weaker dynamical stability under continuous resource supply.

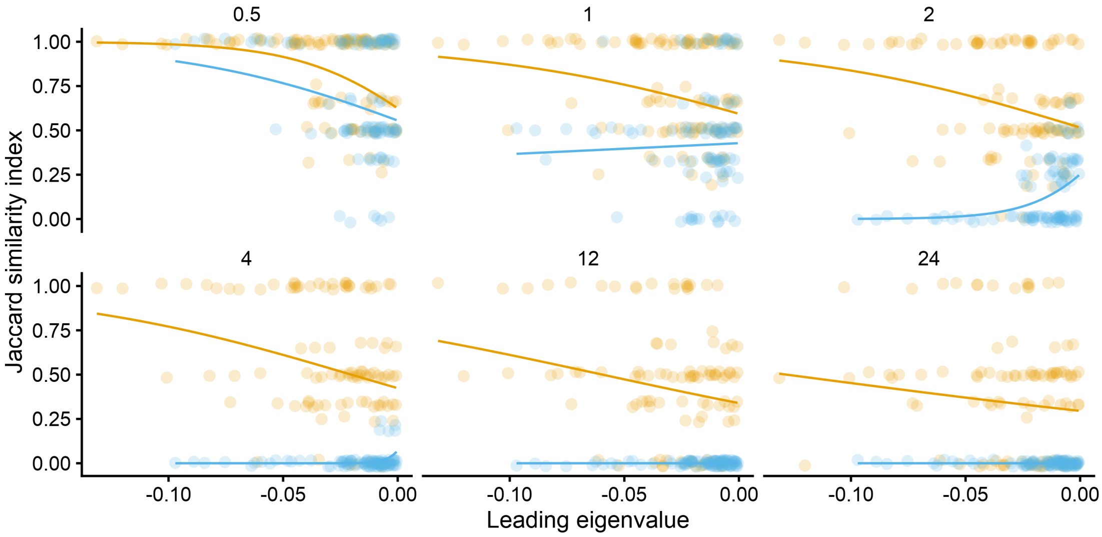

Figure S9: The relationship between compositional overlap and dynamical stability at equilibrium in continuous culture. Larger (less negative) leading eigenvalues indicate less stable systems. Lines of best fit obtained via logistic regression, where a species presence under both pulsed and continuous resource dynamic is treated as a Bernoulli trial. Orange lines and points denote model parametrisations with random sampling of both μ_{max} and K_s; blue lines and points denote model parametrisations with a trade-off imposed between μ_{max} and resource affinity ($\frac{\mu_{max}}{K_s}$). Small points (jittered for clarity) correspond to an individual simulation/solution.

SI-5: Illustrating cycles of growth, saturation and punctuated mortality over consecutive pulses

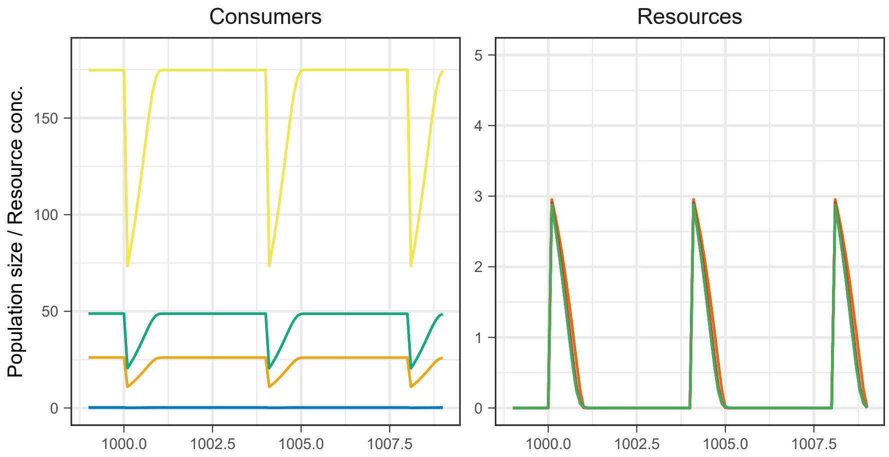

Figure S10: Consumer and resource dynamics from Fig 1b zoomed in over a 10-hour window (pulsing every four hours).

References

[1] Smith, H. L. Bacterial competition in serial transfer culture. *Mathematical Biosciences* **229**, 149–159 (2011).

[2] Letten, A. D. rescomp: An r package for defining, simulating and visualizing ode models of consumer-resource interactions. *bioRxiv* (2022).

[3] Chase, J. & Leibold, M. *Ecological Niches: Linking Classical and Contemporary Approaches* (University of Chicago Press, Chicago, IL, 2003).

[4] Letten, A. D., Ke, P.-J. & Fukami, T. Linking modern coexistence theory and contemporary niche theory. *Ecological Monographs* **87**, 161–177 (2017).

[5] de Mazancourt, C. & Dieckmann, U. Trade-off geometries and frequency-dependent selection. *The American Naturalist* **164**, 765–778 (2004).

[6] Meurer, A. *et al.* Sympy: symbolic computing in python. *PeerJ Computer Science* **3**, e103 (2017).
